# Supplementary material for: HIVprotI: an integrated web based platform for prediction and design of HIV proteins inhibitors
Source: J Cheminform. 2018 Mar 9;10:12. doi: 10.1186/s13321-018-0266-y (PMC5845081; doi:10.1186/s13321-018-0266-y)
Supplement: Supplementary file 2 — Additional file 2. Source code of HIVProtI web server. [file 13321_2018_266_MOESM2_ESM.zip › HIVprotI_Source-code/hivprotI/weka-3-6-6/doc/weka/filters/unsupervised/attribute/AddValues.html]

AddValues


---


|  |  |  |
| --- | --- | --- |
| **Overview** \| **Package** \| **Class** \| **Tree** \| **Deprecated** \| **Index** \| **Help** \| **Weka's home** | |  |
| **PREV CLASS**   **NEXT CLASS** | **FRAMES**    **NO FRAMES**     **All Classes** |  |
| SUMMARY: NESTED \| FIELD \| CONSTR \| METHOD | DETAIL: FIELD \| CONSTR \| METHOD |  |


---


## weka.filters.unsupervised.attribute Class AddValues

```
java.lang.Object
  weka.filters.Filter
      weka.filters.unsupervised.attribute.AddValues
```

**All Implemented Interfaces:**: java.io.Serializable, CapabilitiesHandler, OptionHandler, RevisionHandler, StreamableFilter, UnsupervisedFilter

---

``` public class AddValues extends Filter implements UnsupervisedFilter, StreamableFilter, OptionHandler ```

Adds the labels from the given list to an attribute if they are missing. The labels can also be sorted in an ascending manner. If no labels are provided then only the (optional) sorting applies.


Valid options are:

```
 -C <col>
  Sets the attribute index
  (default last).
```

```
 -L <label1,label2,...>
  Comma-separated list of labels to add.
  (default: none)
```

```
 -S
  Turns on the sorting of the labels.
```

Based on code from AddValues.

**Version:**
:   $Revision: 6897 $

**Author:**
:   FracPete (fracpete at waikato dot ac dot nz)

**See Also:**: `AddValues`, Serialized Form

---

**Constructor Summary**

`AddValues()`


| **Method Summary** | |
| --- | --- |
| `java.lang.String` | `attributeIndexTipText()`             Returns the tip text for this property |
| `java.lang.String` | `getAttributeIndex()`             Get the index of the attribute used. |
| `Capabilities` | `getCapabilities()`             Returns the Capabilities of this filter. |
| `java.lang.String` | `getLabels()`             Get the comma-separated list of labels that are added. |
| `java.lang.String[]` | `getOptions()`             Gets the current settings of the filter. |
| `java.lang.String` | `getRevision()`             Returns the revision string. |
| `boolean` | `getSort()`             Gets whether the labels are sorted or not. |
| `java.lang.String` | `globalInfo()`             Returns a string describing this filter |
| `boolean` | `input(Instance instance)`             Input an instance for filtering. |
| `java.lang.String` | `labelsTipText()`             Returns the tip text for this property |
| `java.util.Enumeration` | `listOptions()`             Returns an enumeration describing the available options. |
| `static void` | `main(java.lang.String[] args)`             Main method for testing and running this class. |
| `void` | `setAttributeIndex(java.lang.String attIndex)`             Sets index of the attribute used. |
| `boolean` | `setInputFormat(Instances instanceInfo)`             Sets the format of the input instances. |
| `void` | `setLabels(java.lang.String value)`             Sets the comma-separated list of labels. |
| `void` | `setOptions(java.lang.String[] options)`             Parses a given list of options. |
| `void` | `setSort(boolean value)`             Sets whether the labels are sorted. |
| `java.lang.String` | `sortTipText()`             Returns the tip text for this property |

**Methods inherited from class weka.filters.Filter**

`batchFilterFile, batchFinished, filterFile, getCapabilities, getOutputFormat, isFirstBatchDone, isNewBatch, isOutputFormatDefined, makeCopies, makeCopy, numPendingOutput, output, outputPeek, toString, useFilter, wekaStaticWrapper`

**Methods inherited from class java.lang.Object**

`equals, getClass, hashCode, notify, notifyAll, wait, wait, wait`

**Constructor Detail**

### AddValues

```
public AddValues()
```


**Method Detail**

### globalInfo

```
public java.lang.String globalInfo()
```

:   Returns a string describing this filter

    :   **Returns:**: a description of the filter suitable for displaying in the explorer/experimenter gui

---


### listOptions

```
public java.util.Enumeration listOptions()
```

:   Returns an enumeration describing the available options.

    :   **Specified by:**: `listOptions` in interface `OptionHandler`
    :   **Returns:**: an enumeration of all the available options.

---


### setOptions

```
public void setOptions(java.lang.String[] options)
                throws java.lang.Exception
```

:   Parses a given list of options.


    Valid options are:

    ```
     -C <col>
      Sets the attribute index
      (default last).
    ```

    ```
     -L <label1,label2,...>
      Comma-separated list of labels to add.
      (default: none)
    ```

    ```
     -S
      Turns on the sorting of the labels.
    ```

    :   **Specified by:**: `setOptions` in interface `OptionHandler`
    :   **Parameters:**: `options` - the list of options as an array of strings **Throws:**: `java.lang.Exception` - if an option is not supported

---


### getOptions

```
public java.lang.String[] getOptions()
```

:   Gets the current settings of the filter.

    :   **Specified by:**: `getOptions` in interface `OptionHandler`
    :   **Returns:**: an array of strings suitable for passing to setOptions

---


### getCapabilities

```
public Capabilities getCapabilities()
```

:   Returns the Capabilities of this filter.

    :   **Specified by:**: `getCapabilities` in interface `CapabilitiesHandler` **Overrides:**: `getCapabilities` in class `Filter`
    :   **Returns:**: the capabilities of this object **See Also:**: `Capabilities`

---


### setInputFormat

```
public boolean setInputFormat(Instances instanceInfo)
                       throws java.lang.Exception
```

:   Sets the format of the input instances.

    :   **Overrides:**: `setInputFormat` in class `Filter`
    :   **Parameters:**: `instanceInfo` - an Instances object containing the input instance structure (any instances contained in the object are ignored - only the structure is required). **Returns:**: true if the outputFormat may be collected immediately **Throws:**: `java.lang.Exception` - if the input format can't be set successfully

---


### input

```
public boolean input(Instance instance)
```

:   Input an instance for filtering. The instance is processed
    and made available for output immediately.

    :   **Overrides:**: `input` in class `Filter`
    :   **Parameters:**: `instance` - the input instance **Returns:**: true if the filtered instance may now be collected with output(). **Throws:**: `java.lang.IllegalStateException` - if no input format has been set.

---


### attributeIndexTipText

```
public java.lang.String attributeIndexTipText()
```

:   Returns the tip text for this property

    :   **Returns:**: tip text for this property suitable for displaying in the explorer/experimenter gui

---


### getAttributeIndex

```
public java.lang.String getAttributeIndex()
```

:   Get the index of the attribute used.

    :   **Returns:**: the index of the attribute

---


### setAttributeIndex

```
public void setAttributeIndex(java.lang.String attIndex)
```

:   Sets index of the attribute used.

    :   **Parameters:**: `attIndex` - the index of the attribute

---


### labelsTipText

```
public java.lang.String labelsTipText()
```

:   Returns the tip text for this property

    :   **Returns:**: tip text for this property suitable for displaying in the explorer/experimenter gui

---


### getLabels

```
public java.lang.String getLabels()
```

:   Get the comma-separated list of labels that are added.

    :   **Returns:**: the list of labels

---


### setLabels

```
public void setLabels(java.lang.String value)
```

:   Sets the comma-separated list of labels.

    :   **Parameters:**: `value` - the list

---


### sortTipText

```
public java.lang.String sortTipText()
```

:   Returns the tip text for this property

    :   **Returns:**: tip text for this property suitable for displaying in the explorer/experimenter gui

---


### getSort

```
public boolean getSort()
```

:   Gets whether the labels are sorted or not.

    :   **Returns:**: true if the labels are sorted

---


### setSort

```
public void setSort(boolean value)
```

:   Sets whether the labels are sorted.

    :   **Parameters:**: `value` - if true the labels are sorted

---


### getRevision

```
public java.lang.String getRevision()
```

:   Returns the revision string.

    :   **Specified by:**: `getRevision` in interface `RevisionHandler` **Overrides:**: `getRevision` in class `Filter`
    :   **Returns:**: the revision

---


### main

```
public static void main(java.lang.String[] args)
```

:   Main method for testing and running this class.

    :   **Parameters:**: `args` - should contain arguments to the filter: use -h for help


---


|  |  |  |
| --- | --- | --- |
| **Overview** \| **Package** \| **Class** \| **Tree** \| **Deprecated** \| **Index** \| **Help** \| **Weka's home** | |  |
| **PREV CLASS**   **NEXT CLASS** | **FRAMES**    **NO FRAMES**     **All Classes** |  |
| SUMMARY: NESTED \| FIELD \| CONSTR \| METHOD | DETAIL: FIELD \| CONSTR \| METHOD |  |


---
